# Draft genome of three isolates of *Listeria monocytogenes* isolated from *Stenella coeruleoalba* in Italy

**DOI:** 10.1128/mra.01221-23

**Published:** 2024-02-28

**Authors:** Fabio Scarpa, Carla Grattarola, Alessandra Arillo, Virginia Mattioda, Camilla Testori, Giuliana Terracciano, Matteo Senese, Federica Giorda, Simona Zoppi, Daria Sanna, Marco Casu, Simone Peletto

**Affiliations:** 1 Department of Biomedical Sciences, University of Sassari, Sassari, Italy; 2 Istituto Zooprofilattico Sperimentale del Piemonte, Liguria e Valle d’Aosta, Turin, Italy; 3 Istituto Zooprofilattico Sperimentale del Lazio e della Toscana M. Aleandri, Pisa, Italy; 4 Department of Veterinary Medicine, University of Sassari, Sassari, Italy; University of Maryland School of Medicine, Baltimore, Maryland, USA

**Keywords:** genomes, bacteria, bioinformatics, Next-generation sequencing (NGS)

## Abstract

*Listeria monocytogenes* is the etiological agent of the listeriosis. Here, we described three draft genome sequences of *L. monocytogenes* isolated in Italy from stranded individuals of the striped dolphin *Stenella coeruleoalba*. All the genomes have been molecular typed through the multilocus sequence typing to identify the phylogenetic lineage, clonal complex, sublineage, and serogroup.

## ANNOUNCEMENT

*Listeria monocytogenes* is a gram-positive, rod-shaped bacteria that can cause a serious foodborne illness known as listeriosis. Here, we present three draft genome sequences of *L. monocytogenes* obtained from the central nervous system of three individuals of striped dolphin *Stenella coeruleoalba* found in Italy (see Table 1 for details). *Listeria monocytogenes* was detected during routine analysis following the detection protocol for stranded marine mammals (1). Selective cultivation was performed under aerobic conditions by enrichment step in liquid media (1/10 p/v) (FRASER broth, at 30°C for 48 h), followed by subculture on solid media (LSM “Luria-Bertani Solid Medium” and ALOA “Agar Leptospira Medium”) at 37°C for 48 h. Confirmation of *L. monocytogenes* was achieved using the miniaturized biochemical test API Listeria, bioMerieux. The detected strains were subsequently cultured on sheep blood agar (AS) as pure cultures at 37°C for 24–48 h, in preparation for sequencing. Genomic DNA was isolated using Maxwell 16 Cell DNA Purification Kit, according to the manufacturer’s instructions (Promega, Madison, WI, USA), as described by Sévellec et al. (2). The genome was sequenced using Whole-Genome Shotgun conducted by the Istituto Zooprofilattico Sperimentale del Piemonte, Liguria, and Valle d’Aosta. DNA processing was performed using the Nextera XT DNA Library Preparation Kit from Illumina, following the manufacturer’s instructions. The final library was sequenced on an Illumina MiSeq platform with the MiSeq Reagent Kit v3-600 in a 300-bp paired-end run. The MiSeq runs generated a data output of 11.33 Gbp with 77.48% of bases with *Q*-score of 30 or higher, and 92.46% of clusters passing filter. The resulting FASTQ files (see Table 1 for technical details) underwent quality assessment using the FastQC tool within the command line package FASTX-Toolkit v0.7 (3). This toolkit was also utilized for the removal of adapter sequences (command line: fastx_clipper -e 0.1 a CTGTCTCTTATA -l 15) and the trimming of sequences that were too short or of low quality (command line: fastq_quality_filter -v -m 30 -Q 33 -q 25 p 50). Following the workflow described in Scarpa et al. (4), a *de novo* assembly was carried out using SPAdes v3.13.0 (5) with the following command line parameters: spades.py --threads 12 -o . --dataset spades_yaml_file.txt -m 128 --only-assembler.

**TABLE 1:** Details on the whole genomes of *Listeria monocytogenes* and results of MLST typing analyses

Isolates			
	S1_28608	S2_28615	S3_28616
Tissue	Brain	Brain	Brain
Sampling locality	Italy: Tuscany	Italy: Tuscany	Italy: Tuscany
Number of reads^*a*^	3,959,427	5,851,855	3,218,053
Number of nucleotides	940,633,673	2,860,454,848	1,561,701,320
No. of contigs	24	41	20
GC content (%)	37.92%	38.24%	37.85%
Contig L50	2	2	1
Contig N50 (nt)	510,261	510,261	1,519,114
Contig L90	6	6	5
Contig N90 (nt)	223,346	223,346	103,226
Genome length (nt)	2,883,903	2,922,149	3,030,248
No. of predicted CDS	2,822	2,851	2,994
No. of tRNAs	58	59	48
No. of rRNAs	10	12	3
Phylogenetic lineage	I	I	II
Clonal complex	CC6	CC6	CC121
Sublineage	24	41	19
CGMLST type	CT14418	CT14418	CT14417
ST	6	6	121
abcZ	3	3	7
bglA	9	9	6
cat	9	9	8
dapE	3	3	8
dat	3	3	6
ldh	1	1	37
lhkA	5	5	1
Serogroup	IVb	IVb	IIa
GenBank accession number	JAWLVB000000000	JAWLVC000000000	JAWLVD000000000
BioSample accession number	SAMN37915372	SAMN37915373	SAMN37915374
Sequence read archive	SRR26459687	SRR26459686	SRR26459685

^*a*^ The indicated number of reads represents the amount during the pre-filtering step.

The estimated coverage for all sequenced genomes was ensured to be greater than or equal to 100×. Details for statistics on genome assembly are listed in Table 1.

The entire draft genomes were annotated using the NCBI Prokaryotic Genome Annotation Pipeline v4.2 (6) with the best-placed reference protein set method and GeneMarkS-2.

The molecular typing was performed through the multilocus sequence typing using BIGSdb-Lm implemented in *Listeria monocytogenes* web page of the Institute Pasteur (https://bigsdb.pasteur.fr/listeria/) (7) (see Table 1 for results). Default parameters were used for all software unless otherwise specified.

## Data Availability

The complete genome sequences of *Listeria monocytogenes* have been made available through the NCBI GenBank with the following identifiers: BioProject accession number PRJNA1030755, BioSample accession numbers SAMN37915372, SAMN37915373, SAMN37915374, and GenBank genome accession JAWLVB000000000, JAWLVC000000000, and JAWLVD000000000. The original sequence reads used for assembly can be found in the Sequence Read Archive (SRA) under accession numbers SRR26459687, SRR26459686, SRR26459685.
